# Antidepressant Mechanisms of L-Theanine in Tea Based on Network Pharmacology, Molecular Docking, and Molecular Dynamics Simulations

**DOI:** 10.3390/foods15030555

**Published:** 2026-02-04

**Authors:** Yutao Shi, Yuan Yang, Xi Cheng, Canyang Huang, Yan Huang, Li Lu, Shuyan Wang, Yucheng Zheng, Feiquan Wang, Bo Zhang, Shulin Zheng

**Affiliations:** Tea Science Research Institute, Tea Engineering Research Center of Fujian Higher Education, College of Tea and Food Sciences, Wuyi University, Wuyishan 354300, China; 15208608905@163.com (Y.Y.); wyxychengxi@wuyiu.edu.cn (X.C.); 18359531028@163.com (C.H.); yanhuangfst@wuyiu.edu.cn (Y.H.); lilyluwyu@163.com (L.L.); wangshuyan@wuyiu.edu.cn (S.W.); 18094159524@163.com (Y.Z.); wfq1982@wuyiu.edu.cn (F.W.); 13859090163@163.com (B.Z.)

**Keywords:** target prediction, protein–protein interaction (PPI), hub targets, synaptic transmission, binding free energy

## Abstract

L-theanine is a bioactive non-protein amino acid predominantly derived from tea plants (*Camellia sinensis*), widely recognized for its potential benefits in mood regulation and psychological health. Despite its promising neuropsychological profile, the specific molecular targets and mechanisms underlying its antidepressant activity remain incompletely understood. In the present study, an integrated network pharmacology strategy, combined with molecular docking and molecular dynamics (MD) simulations, was employed to systematically elucidate the potential antidepressant mechanisms of L-theanine. By intersecting predicted drug targets with depression-related genes, 40 potential targets were identified. Protein–protein interaction (PPI) network analysis subsequently pinpointed five hub targets: PRKACA, GRIA2, GRIN1, GRIA1, and HTR1A. Functional enrichment analyses (KEGG and GO) indicated that these targets are primarily implicated in critical pathological processes of depression, including neurotransmitter regulation, glutamatergic synaptic transmission, stress response signaling, and neurotrophin-related pathways. Molecular docking revealed favorable binding affinities between L-theanine and the key targets. Furthermore, MD simulations and binding free energy calculations corroborated the structural stability and thermodynamic favorability of these protein–ligand complexes. Overall, this study provides hypothesis-generating insights into the antidepressant mechanisms of L-theanine from a multi-target perspective, offering a theoretical foundation to guide future experimental validation in depression research.

## 1. Introduction

Depression is a pervasive mental disorder that severely impacts the psychological well-being of millions of individuals worldwide. In recent years, the prevalence of depression has continued to escalate, establishing it as a critical global public health concern [1]. Clinical manifestations typically include persistent low mood, anhedonia, fatigue, psychomotor retardation, and cognitive impairments [2]. Currently, the primary therapeutic modalities encompass psychotherapy and pharmacotherapy. Guidelines from the American Psychological Association recommend prioritizing psychotherapy for adolescent depression, reserving pharmacological agents like fluoxetine for severe cases [3]. Similarly, the National Institute for Health and Care Excellence (NICE) in the United Kingdom advises against the routine use of antidepressants for mild depression, suggesting that pharmacotherapy be considered for moderate to severe cases only after psychotherapeutic interventions have proven insufficient [4]. Although available antidepressants demonstrate efficacy in alleviating symptoms, their clinical utility is often limited by adverse side effects, delayed onset of action, and substantial interindividual variability in therapeutic response [5,6]. Consequently, there is an urgent need to develop safer and more effective preventive and therapeutic strategies.

**Figure 1:**
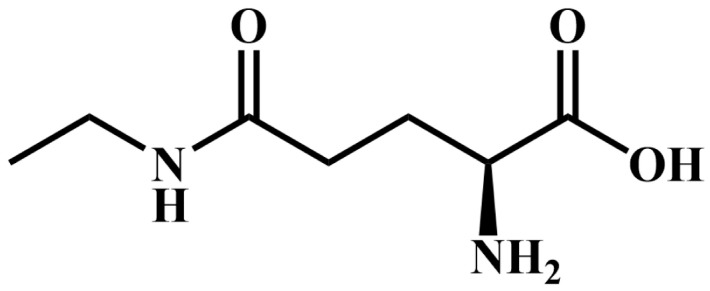
Chemical structure of L-theanine.

L-theanine is a characteristic bioactive non-protein amino acid predominantly derived from tea plants (*Camellia sinensis*), although it has also been identified in specific fungal species such as *Boletus badius* and *Hericium erinaceus*. Its chemical structure is illustrated in Figure 1. Beyond functioning as a key determinant of the umami and sweet flavor profile, L-theanine is widely recognized as a pivotal bioactive constituent of tea [7]. Recently, L-theanine has garnered increasing attention due to its diverse pharmacological activities, particularly its beneficial effects on psychological health, including stress reduction and anxiolysis [8,9]. Mechanistically, L-theanine has been reported to modulate depression-related neurobiology by regulating brain neurotransmitter levels, such as dopamine and serotonin (5-hydroxytryptamine) [10]. In animal models, L-theanine supplementation has been shown to upregulate brain-derived neurotrophic factor (BDNF) and dopamine through pathways involving platelet factor 4 and G protein subunit αi2 [11]. Furthermore, clinical evidence indicates that adjunctive administration of L-theanine (250 mg/day for 8 weeks) to patients with major depressive disorder is safe and confers multiple benefits, including the amelioration of depressive symptoms, anxiety, sleep disturbances, and cognitive dysfunction [12]. Given its favorable safety profile and therapeutic potential, L-theanine represents a promising candidate for the development of novel antidepressant interventions.

Despite accumulating evidence supporting the antidepressant potential of L-theanine, its specific molecular targets and underlying mechanisms of action remain incompletely understood. Network pharmacology, a systems biology-based approach, enables the integration of multidimensional data encompassing compounds, targets, pathways, and diseases, facilitating a holistic analysis of the multi-target and multi-pathway characteristics of bioactive compounds [13]. Complementarily, molecular docking and molecular dynamics (MD) simulations allow for the detailed evaluation of ligand–protein interactions by predicting binding modes, affinities, and the structural stability of the resulting complexes [14,15]. Accordingly, in the present study, an integrated strategy combining network pharmacology with molecular docking and MD simulations was employed to systematically elucidate the potential targets and signaling pathways associated with the antidepressant effects of L-theanine. From a multi-target and multi-pathway perspective, this study aims to provide computational evidence and hypothesis-generating insights, thereby establishing a theoretical foundation for future experimental investigations of tea-derived functional components in depression research.

## 2. Materials and Methods

### 2.1. Collection of L-Theanine-Related Targets

The SMILES structural formula of L-theanine was obtained from the PubChem database (https://pubchem.ncbi.nlm.nih.gov/, accessed on 15 August 2025) [16]. Using this SMILES string as the input, potential targets of L-theanine were predicted independently via the SEA Search Server (https://sea.bkslab.org/, accessed on 15 August 2025), SwissTargetPrediction (http://www.swisstargetprediction.ch/, accessed on 15 August 2025), Super-PRED (https://prediction.charite.de/index.php, accessed on 15 August 2025), and CODD-Pred (http://codd.iddd.group/, accessed on 15 August 2025) databases [17,18,19]. Targets derived from the four databases were integrated, and standardized gene names were obtained using the UniProt database (https://www.uniprot.org/, accessed on 15 August 2025). Invalid entries, non-human targets, and duplicate targets were removed. Subsequently, a L-theanine–target interaction network was constructed and visualized using Cytoscape (v3.9.1) software.

### 2.2. Screening of Depression-Related Targets

Depression-related targets were retrieved by searching the DisGeNET (https://www.disgenet.org/, accessed on 15 August 2025), OMIM (https://omim.org/, accessed on 15 August 2025), and GeneCards (https://www.genecards.org/, accessed on 15 August 2025) disease databases using the keyword “Depression” [14]. In the DisGeNET database, targets with a score_gda ≥ 0.1 were retained, whereas in the GeneCards database, targets with a relevance score ≥ 5 were selected [20]. All gene entries associated with depression in the OMIM database were included. Targets obtained from the three databases were merged, and duplicate entries were removed to generate a candidate set of depression-related targets.

### 2.3. Identification of Key Targets and PPI Network

To explore the potential key targets involved in the antidepressant effects of L-theanine, the predicted targets of L-theanine were intersected with the depression-related targets, and a Venn diagram was generated using an online platform (https://cloud.metware.cn/, accessed on 16 August 2025). The resulting 40 overlapping targets were imported into the STRING database (http://string-db.org/, accessed on 16 August 2025) to construct a protein–protein interaction (PPI) network [21]. The species was set to “Human”, and the minimum required interaction score (combined score) was defined as 0.4, with all other parameters set to default values. The PPI network data exported from STRING were then imported into Cytoscape (v3.9.1) software. Network topology parameters were calculated using the NetworkAnalyzer plugin (v4.4.8), and key targets associated with the antidepressant activity of L-theanine were screened based on node degree values.

### 2.4. KEGG and GO Pathway Enrichment Analyses

To further elucidate the biological functions of the key target genes, Gene Ontology (GO) functional annotation and Kyoto Encyclopedia of Genes and Genomes (KEGG) pathway enrichment analyses were performed for the overlapping targets using the “clusterProfiler” package (v4.8.1) in the R software (v4.3.2) environment, with a significance threshold of qvalue (FDR) < 0.05. KEGG pathway enrichment analysis was applied to identify the key signaling pathways associated with the antidepressant targets of L-theanine. GO analysis was conducted at three levels, including biological process (BP), cellular component (CC), and molecular function (MF), to comprehensively characterize the functional attributes of the targets. The results of KEGG and GO enrichment analyses were visualized using an online tool (https://cloud.metware.cn/, accessed on 18 August 2025).

### 2.5. Molecular Docking

The ligand file of L-theanine in SDF format was downloaded from the PubChem database (https://pubchem.ncbi.nlm.nih.gov/, accessed on 20 August 2025). The UniProt accession numbers of PRKACA, GRIA2, GRIN1, GRIA1, and HTR1A were obtained from the UniProt database (https://www.uniprot.org/, accessed on 20 August 2025). The corresponding three-dimensional protein structures were subsequently downloaded from the Protein Data Bank (PDB) or the AlphaFold Protein Structure Database, including PRKACA (PDB ID: 3AMA), GRIA2 (PDB ID: 2WJW), GRIN1 (AlphaFold ID: AF-Q05586-F1), GRIA1 (AlphaFold ID: AF-P42261-F1), and HTR1A (AlphaFold ID: AF-P08908-F1). Molecular docking between L-theanine and the above protein receptors was performed using AutoDock Vina (v1.2.5) [22] to evaluate binding modes and binding energies. The docking results were further analyzed and visualized using Discovery Studio (v2019) software.

### 2.6. Molecular Dynamics Simulations

Molecular dynamics (MD) simulations were performed using GROMACS (v2025.1) to investigate the ligand–protein complexes of L-theanine with five key targets: PRKACA, GRIA2, GRIN1, GRIA1, and HTR1A. The systems were parameterized using the AMBER99SB force field and solvated with the SPC water model. Na^+^ and Cl^−^ ions were introduced to neutralize the system and adjust the ionic strength to 0.15 M. Prior to the production runs, the systems underwent energy minimization using the steepest descent algorithm, followed by equilibration under NVT and NPT ensembles. Production MD simulations were conducted for 100 ns with a 2 fs time step. During simulations, the temperature was maintained at 300 K using the V-rescale thermostat (τ_t_ = 0.1 ps), and pressure was controlled isotropically at 1.0 atm via the Berendsen barostat (τ_p_ = 2.0 ps). Trajectories were recorded every 50 ps [23]. Key trajectory parameters, including root mean square deviation (RMSD), root mean square fluctuation (RMSF), radius of gyration (Rg), solvent-accessible surface area (SASA), and hydrogen bond counts, were evaluated and plotted with GraphPad Prism (v9.5.1) [24]. In addition, free energy landscapes were constructed and analyzed based on RMSD, Rg, and Gibbs free energy, and visualized using Origin (v2024) software.

## 3. Results

### 3.1. Screening of Potential Targets of L-Theanine Against Depression

**Figure 2:**
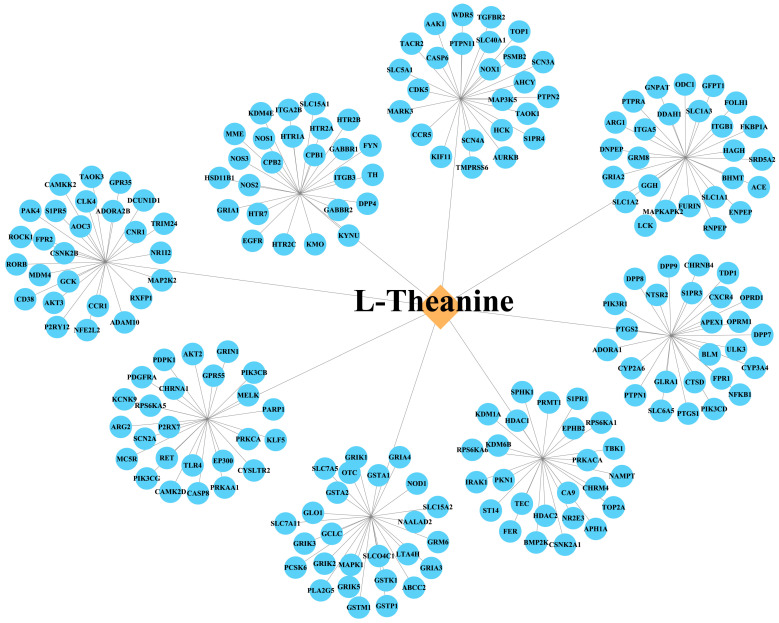
Construction of the L-theanine–target interaction network.

Potential targets of L-theanine were predicted using the SEA Search Server, SwissTargetPrediction, Super-PRED, and CODD-Pred databases, yielding 49, 34, 123, and 16 targets, respectively. After integration of the results from the four databases and removal of duplicate entries, a total of 204 candidate targets of L-theanine were obtained. A L-theanine–target interaction network was subsequently constructed using Cytoscape (v3.9.1) (Figure 2; Table S1). Meanwhile, a total of 558 depression-related targets were collected from the DisGeNET, GeneCards, and OMIM databases, including 346 targets from DisGeNET, 358 from GeneCards, and 3 from OMIM (Table S2). After elimination of redundant entries, a final candidate set of depression-related targets was generated.

### 3.2. Identification of Key Targets of L-Theanine Against Depression

**Figure 3:**
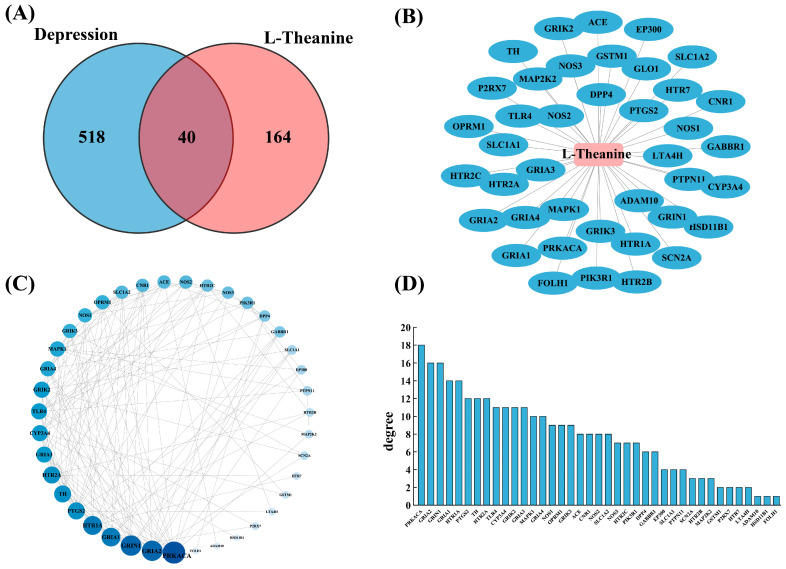
(**A**) Venn diagram showing the intersection between predicted targets of L-theanine and depression-related targets. (**B**) L-theanine–overlapping target interaction network. (**C**) PPI network of the 40 overlapping targets. (**D**) Node degree values of the 40 overlapping targets in the PPI network.

The predicted targets of L-theanine were intersected with the depression-related targets, resulting in 40 common targets (Figure 3A). A L-theanine–common target interactioin network was then constructed and visualized using Cytoscape (Figure 3B). These 40 overlapping targets were further imported into the STRING database to construct a PPI network, which was subsequently visualized in Cytoscape (Figure 3C). One target without interaction information was excluded by the STRING database, and the final PPI network consisted of 39 nodes and 302 edges. The degree value of each node was used to evaluate its topological importance within the network. The targets with the highest degree values were PRKACA (18), GRIA2 (16), GRIN1 (16), GRIA1 (14), HTR1A (14), followed by PTGS2 (12), TH (12), HTR2A (12), TLR4 (11), CYP3A4 (11), GRIK2 (11), and GRIA3 (11) (Figure 3D). Based on degree values, PRKACA, GRIA2, GRIN1, GRIA1, and HTR1A were identified as the top five hub targets and selected for subsequent analyses.

### 3.3. KEGG and GO Pathway Enrichment Results

**Figure 4:**
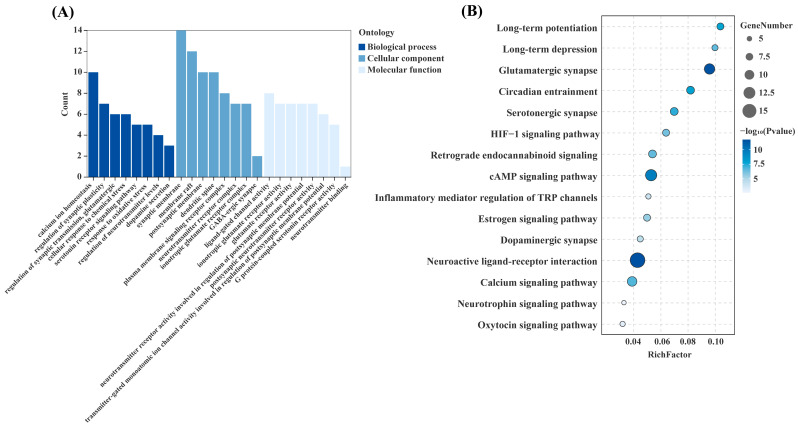
(**A**) KEGG pathway enrichment analysis of the 40 key targets. (**B**) GO enrichment analysis of the 40 key targets.

To elucidate the potential antidepressant mechanisms of L-theanine at a systems level, KEGG and GO enrichment analyses were performed for the 40 overlapping targets. KEGG enrichment analysis revealed that these targets were significantly enriched in 129 pathways (FDR < 0.05), which were predominantly associated with nervous system function, signal transduction, and biological rhythms. Notably enriched pathways included neuroactive ligand–receptor interaction (hsa04080), long-term depression (hsa04730), retrograde endocannabinoid signaling (hsa04723), dopaminergic synapse (hsa04728), the HIF-1 signaling pathway (hsa04066), and the neurotrophin signaling pathway (hsa04722) (Figure 4A, Table S3). GO enrichment analysis indicated that the overlapping targets were enriched in 1003 BP terms, 83 CC terms, and 138 MF terms, representing 81.94%, 6.78%, and 11.27% of all enriched GO terms, respectively (FDR < 0.05) (Figure 4B; Table S4). In the BP category, the identified targets were predominantly involved in biological processes related to neural function and stress responses, including regulation of neurotransmitter levels (GO:0001505), modulation of glutamatergic synaptic transmission (GO:0051966), dopamine secretion (GO:0014046), response to oxidative stress (GO:0006979), and maintenance of calcium ion homeostasis (GO:0055074). In the CC category, the identified targets were primarily distributed across synaptic membranes (GO:0097060), plasma membrane signaling receptor complexes (GO:0098802), and neurotransmitter receptor complexes (GO:0098878). In the MF category, the identified targets mainly exhibited molecular functions related to postsynaptic neurotransmitter receptor activity (GO:0098960), transmitter-gated monovalent ion channel activity involved in postsynaptic membrane potential regulation (GO:1904315), ligand-gated channel activity (GO:0022834), and neurotransmitter binding (GO:0042165). Collectively, the KEGG and GO enrichment results suggest that L-theanine may participate in the complex pathological processes of depression through the regulation of neurotransmitter systems, synaptic transmission, key signaling pathways, cellular stress responses, and membrane-associated structures and functions.

### 3.4. Molecular Docking Analysis

**Figure 5:**
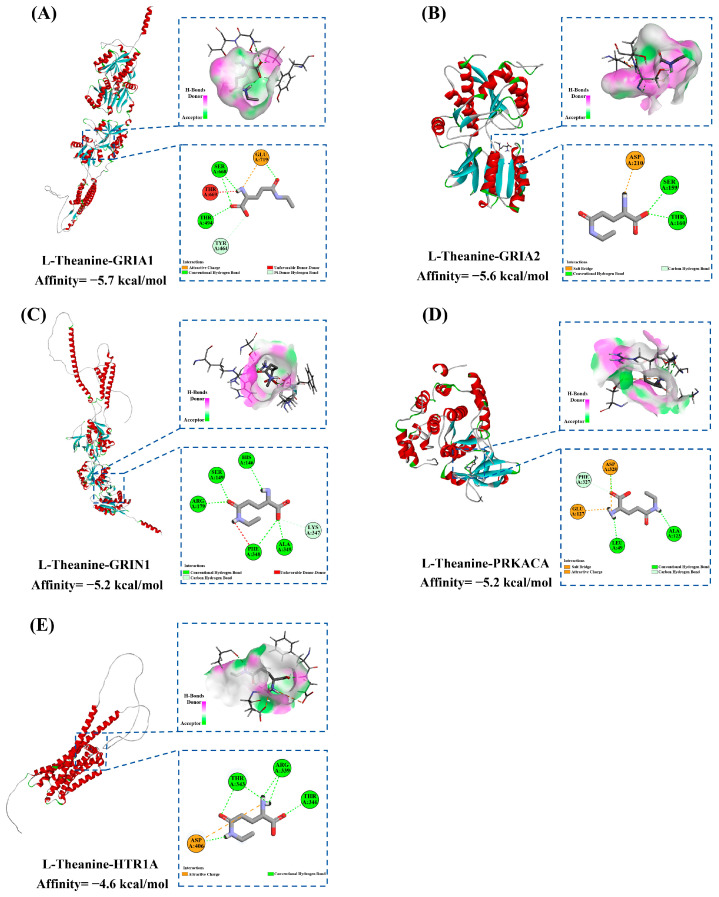
Molecular docking structure between L-theanine and (**A**) GRIA1, (**B**) GRIA2, (**C**) GRIN1, (**D**) PRKACA, and (**E**) HTR1A.

Molecular docking is a widely applied computational technique for estimating the binding conformations and affinities between proteins and small-molecule ligands [25]. In the present study, the five targets with the highest degree values in the PPI network—PRKACA, GRIA2, GRIN1, GRIA1, and HTR1A—were selected as receptors, with L-theanine employed as the ligand for molecular docking analysis (Figure 5, Table S5). The results showed that the docking scores of L-theanine with the five targets ranged from −4.6 to −5.7 kcal/mol. These scores indicate moderate but favorable binding affinities, which are characteristic of small, highly polar molecules like L-theanine. Ranking based on docking scores revealed that the GRIA1–L-theanine complex exhibited the lowest binding energy (docking score = −5.7 kcal/mol), followed by L-theanine–GRIA2 (−5.6 kcal/mol), L-theanine–GRIN1 (−5.2 kcal/mol), L-theanine–PRKACA (−5.2 kcal/mol), and L-theanine–HTR1A (−4.6 kcal/mol). Specifically, in the L-theanine–GRIA1 complex, four hydrogen bonds were formed with ALA123 (2.15 Å), LEU49 (2.29 Å), ASP328 (2.02 Å), and PHE327 (2.68 Å), accompanied by electrostatic interactions with GLU127 (2.15 Å) and ASP328 (2.15 Å) (Figure 5A). In the L-theanine–GRIA2 complex, two hydrogen bonds were observed with SER159 (2.50 Å) and THR160 (2.09 Å), along with an electrostatic interaction with ASP210 (4.47 Å) (Figure 5B). In the L-theanine–GRIN1 complex, six hydrogen bonds were formed with HIS146 (2.56 Å), SER149 (2.20 Å), ARG179 (2.43 Å), PHE348 (2.84 Å), ALA349 (2.04 Å), and LYS347 (3.06 Å), in addition to a repulsive interaction with PHE348 (2.48 Å) (Figure 5C). In the L-theanine–PRKACA complex, five hydrogen bonds were identified with SER668 (2.13 Å and 2.78 Å), THR494 (2.53 Å), GLU719 (2.21 Å), and TYR464 (4.03 Å), together with an electrostatic interaction with GLU719 (3.57 Å) and a repulsive interaction with THR669 (1.86 Å) (Figure 5D). In the L-theanine–HTR1A complex, six hydrogen bonds were formed with ASP406 (2.11 Å), ARG339 (2.78 Å and 2.78 Å), THR343 (2.11 Å and 2.46 Å), and THR346 (2.09 Å), accompanied by an electrostatic interaction with ASP406 (5.28 Å) (Figure 5E).

Overall, the findings suggest that L-theanine exhibits stable binding to the key targets, mainly mediated by hydrogen bonds and electrostatic forces. A detailed understanding of these molecular interactions contributes to elucidating the potential antidepressant mechanisms of L-theanine.

### 3.5. MD Simulations and Binding Free Energy Calculations

**Figure 6:**
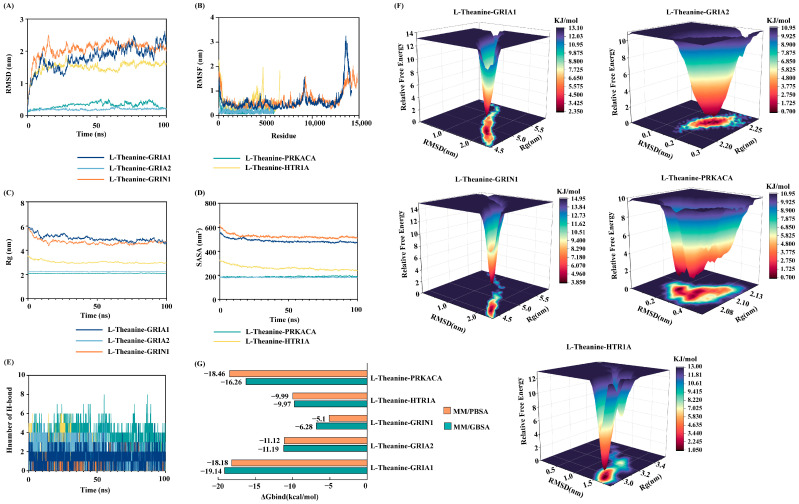
Molecular dynamics simulation and binding free energy analysis of the five protein–ligand complexes. (**A**) RMSD values of the five complexes. (**B**) RMSF values of the five complexes. (**C**) Rg values of the five complexes. (**D**) SASA values of the five complexes. (**E**) Number of hydrogen bonds in the five complexes. The deep blue represents L-theanine–GRIA1; the light blue represents L-theanine–GRIA2; the orange represents L-theanine–GRIN1; the aqua green represents L-theanine–PRKACA; and the pale yellow represents L-theanine–HTR1A. (**F**) Free energy landscapes of the five complexes. (**G**) ΔG_bind_ of the five complexes calculated using the MM/GBSA and MM/PBSA methods.

To further evaluate the dynamic stability and binding characteristics of the protein–ligand complexes, 100 ns MD simulations were performed for five complexes, namely L-theanine–PRKACA, L-theanine–GRIA2, L-theanine–GRIN1, L-theanine–GRIA1, and L-theanine–HTR1A. As shown by the RMSD profiles, all five complexes rapidly reached equilibrium after initial fluctuations during the early stages of the simulations, with average RMSD values stabilizing at 1.80 ± 0.36 Å, 0.20 ± 0.03 Å, 2.06 ± 0.27 Å, 0.32 ± 0.08 Å, and 1.51 ± 0.17 Å, respectively (Figure 6A). These results indicate that the overall conformations of the complexes remained stable during the later stages of the simulations. The fluctuation of amino acid residues was further assessed using the RMSF values [26]. The average RMSF values for the five complexes were stable at 0.17 ± 0.02 nm, 0.14 ± 0.08 nm, 0.62 ± 0.03 nm, 0.57 ± 0.04 nm, and 0.52 ± 0.04 nm, respectively, indicating that the overall conformation of the complexes remained stable (Figure 6B). The compactness of the complexes was evaluated using the Rg. As illustrated in Figure 6C, the Rg values of the five complexes exhibited only minor fluctuations throughout the simulations and stabilized at 4.99 ± 0.26 nm, 2.23 ± 0.01 nm, 4.67 ± 0.11 nm, 2.09 ± 0.01 nm, and 3.02 ± 0.11 nm, respectively, suggesting that the overall structural compactness of the systems was well maintained. The SASA, an important indicator of protein folding status and structural stability, also remained relatively constant during the simulations. The average SASA values of the five complexes were approximately 487.69 ± 15.95 nm^2^, 182.60 ± 2.55 nm^2^, 524.45 ± 17.18 nm^2^, 189.07 ± 3.74 nm^2^, and 262.00 ± 18.16 nm^2^, respectively (Figure 6D). Hydrogen bond analysis revealed noticeable differences in the number of hydrogen bonds among the complexes. The L-theanine–PRKACA complex exhibited the highest hydrogen bond number and density, followed by the L-theanine–GRIA2, L-theanine–GRIA1, L-theanine–HTR1A, and L-theanine–GRIN1 complexes (Figure 6E), indicating that the interaction between L-theanine and PRKACA was relatively stronger. Based on RMSD and Rg values, the relative Gibbs free energy was further calculated, and free energy landscapes were constructed using RMSD, Rg, and Gibbs free energy as the X, Y, and Z axes, respectively, to assess the conformational stability of the complexes in different conformational spaces (Figure 6F). All five complexes displayed distinct low-energy basins, with the red regions corresponding to the lowest free energy states and representing the most stable conformations.

To assess the energetic favorability of protein–ligand interactions, the binding free energies (ΔG_bind_) of the five complexes were estimated using MM/GBSA and MM/PBSA approaches. According to the MM/GBSA calculations, the ΔG_bind_ values were ranked as follows: L-theanine–GRIA1 (−19.14 kcal/mol) < L-theanine–PRKACA (−16.26 kcal/mol) < L-theanine–GRIA2 (−11.19 kcal/mol) < L-theanine–HTR1A (−9.77 kcal/mol) < L-theanine–GRIN1 (−6.82 kcal/mol). Similarly, the MM/PBSA results showed the following ranking: L-theanine–PRKACA (−18.64 kcal/mol) < L-theanine–GRIA1 (−18.18 kcal/mol) < L-theanine–GRIA2 (−11.12 kcal/mol) < L-theanine–HTR1A (−9.99 kcal/mol) < L-theanine–GRIN1 (−5.10 kcal/mol) (Figure 6G). The overall trends of ΔG_bind_ obtained from the two methods were highly consistent, and all values were negative, indicating that the binding of L-theanine to these five targets is thermodynamically favorable. Moreover, complexes with larger absolute ΔG_bind_ values exhibited stronger binding affinities [27]. Taken together, the combined analyses of RMSD, Rg, SASA, hydrogen bond number, and binding free energy demonstrate that L-theanine is capable of forming stable complexes with PRKACA, GRIA2, GRIN1, GRIA1, and HTR1A, exhibiting strong and favorable binding characteristics.

## 4. Discussion

Depression exerts profound effects on both psychological and physical health and has emerged as a major global public health challenge. In recent years, the progressive elucidation of the pharmacological activities of L-theanine has highlighted its potential as a natural bioactive candidate with promising developmental value. Previous studies have demonstrated that L-theanine can improve psychological well-being and alleviate stress and anxiety, thereby contributing to the mitigation of depression-related symptoms to a certain extent [8,9]. On this basis, the present study integrated network pharmacology with molecular docking and molecular dynamics simulations to construct a comprehensive L-theanine–target–disease network, aimed at systematically exploring the key targets and potential molecular mechanisms underlying the antidepressant effects of L-theanine.

Through PPI network analysis, five key targets potentially mediating the antidepressant activity of L-theanine were identified: PRKACA, GRIA2, GRIN1, GRIA1, and HTR1A. PRKACA, the catalytic subunit of protein kinase A (PKA), acts as a critical downstream regulator in multiple neurotransmitter signaling pathways. Previous research indicates that PRKACA expression is significantly downregulated in chronic unpredictable mild stress (CUMS)–induced depression-like rat models, and its upregulation contributes to the improvement of depressive behaviors [28]. GRIA1, GRIA2, and GRIN1 are integral subunits of glutamate receptors. Glutamatergic dysregulation has been extensively implicated in major depressive disorder and other mood disorders [29]. Among these, GRIA2, an AMPA-type glutamate receptor subunit, is closely associated with the pathogenesis of schizophrenia, MDD, and bipolar disorder [30,31]. GRIN1 encodes an essential subunit of the NMDA receptor and is critically involved in the modulation of neuronal excitability and synaptic plasticity, with its dysregulation being implicated in susceptibility to mood disorders [32]. Furthermore, GRIA1 plays a pivotal role in glutamatergic synaptic transmission, and its dysregulation is intricately linked to the pathophysiology of MDD, underscoring its potential as a critical therapeutic target [33]. Additionally, HTR1A (the 5-HT1A receptor) is a classical serotonin receptor subtype central to the pharmacological actions of many antidepressants. The DNA methylation status of the HTR1A gene has been reported to be closely associated with MDD and therapeutic responses to treatment [34]. Collectively, PRKACA, GRIA2, GRIN1, GRIA1, and HTR1A are intimately involved in the pathological processes of depression, suggesting that L-theanine may exert antidepressant effects through the coordinated multi-target modulation of these critical nodes.

GO and KEGG enrichment analyses further delineated the functional and pathway-level framework underlying the potential antidepressant mechanisms of L-theanine. GO enrichment results indicated that the overlapping targets of L-theanine are extensively involved in biological processes such as neurotransmitter regulation, synaptic transmission, oxidative stress responses, and the maintenance of calcium ion homeostasis—processes highly relevant to the pathogenesis of depression. KEGG pathway analysis revealed that these targets are primarily enriched in pathways related to neuroactive ligand–receptor interaction, dopaminergic synapses, long-term depression (LTD), retrograde endocannabinoid signaling, and neurotrophin-related signaling pathways. Notably, these enriched pathways largely overlap with well-established neurobiological processes implicated in depression, suggesting consistency with existing knowledge rather than pathway specificity. Accumulating evidence has shown that imbalances in monoaminergic neurotransmitter systems, including serotonin, norepinephrine, and dopamine, can markedly impair emotional regulation and cognitive function [35]. Dysregulation of synaptic transmission and endocannabinoid signaling may lead to impaired synaptic plasticity and maladaptive stress responses [36,37]. In addition, decreased expression of neurotrophic factors, including brain-derived neurotrophic factor (BDNF), has been linked to degenerative changes in neuronal structure and function [38]. The key targets identified in this study are highly coupled with these pathological processes. Specifically, GRIA1 and GRIA2 directly participate in glutamatergic synaptic transmission and synaptic plasticity regulation, whereas PRKACA modulates neural network function via the cAMP–PKA signaling pathway downstream of multiple receptors [39,40]. GRIN1 is involved in glutamate receptor-mediated synaptic transmission and cellular stress responses [41], while HTR1A serves as a critical regulator within the serotonergic system [42]. Therefore, these enrichment results should be interpreted as functional associations that help prioritize candidate targets/pathways for downstream validation, rather than definitive mechanistic evidence. Overall, L-theanine may potentially influence depression-related disturbances in neurotransmitter homeostasis, synaptic function, and stress responses through these predicted targets, which warrants further validation.

Building upon these findings, molecular docking and MD simulations were employed to assess the binding stability and interaction mechanisms between L-theanine and the key targets. Molecular docking analysis revealed binding energies ranging from −4.6 to −5.7 kcal/mol. These values indicate moderate but favorable binding affinities, consistent with the physicochemical properties of small, polar molecules like L-theanine [43]. Although these energies do not imply the high-potency inhibition typical of synthetic ligands, they suggest that L-theanine can stably occupy the active pockets of these receptors. Structural analysis further highlighted that L-theanine predominantly interacts with key residues through hydrogen bonds and electrostatic interactions, providing a physicochemical basis for specific molecular recognition. To capture the temporal stability of these interactions, MD simulations were conducted. Rather than exhibiting significant conformational drifts, the RMSD, RMSF, Rg, and SASA profiles for all five complexes remained stable throughout the simulation period, indicating that the systems rapidly reached equilibrium and maintained structural compactness [44,45]. Notably, the consistent hydrogen bond occupancy observed during the trajectories underscores the good structural compatibility between L-theanine and the target proteins. Furthermore, MM/GBSA and MM/PBSA calculations demonstrated consistent negative binding free energies, substantiating that the formation of these complexes is thermodynamically favorable. These dynamic energetic profiles corroborate the molecular docking results, providing robust computational support for the multi-target mechanisms predicted by network pharmacology.

In summary, by integrating network pharmacology, GO and KEGG enrichment analyses, molecular docking, and molecular dynamics simulations, this study systematically elucidated the potential antidepressant mechanisms of L-theanine from multi-target, multi-pathway, and multi-scale perspectives. L-theanine is hypothesized to exert antidepressant effects by targeting key proteins, including PRKACA, GRIA2, GRIN1, GRIA1, and HTR1A. This multi-target interaction profile is predicted to modulate critical biological processes—such as glutamatergic synaptic transmission, monoaminergic signaling, stress responses, and neurotrophin-related pathways—thereby theoretically contributing to the amelioration of depression-associated neurobiological dysfunctions. Nevertheless, several limitations should be acknowledged. First, the present study is primarily based on public databases and computational simulations; thus, the findings are predictive and hypothesis-driven, lacking experimental validation at the cellular and animal levels. Second, differences in data update frequency and inclusion criteria among databases may have influenced target screening outcomes. Third, although L-theanine is widely consumed as a dietary component and is generally regarded as safe, this study did not include a dedicated ADMET and toxicity assessments, which limits the translational interpretation of the computational findings. Future work should incorporate systematic ADMET and safety evaluations together with experimental validation. Future studies should build upon the key targets and pathways identified herein by conducting in vitro and in vivo functional experiments to validate the predicted regulatory effects of L-theanine on PRKACA, GRIA2, GRIN1, GRIA1, and HTR1A, and to further assess their potential roles in depression-like behaviors. In addition, the integration of multi-omics approaches, such as transcriptomics and metabolomics, may facilitate the construction of a more comprehensive mechanistic network, thereby providing additional support for future research on natural products in depression-related intervention.

## 5. Conclusions

This study elucidates the antidepressant effects of L-theanine by identifying key targets and molecular mechanisms through an integrated network-based and computational analysis. Network analysis identified PRKACA, GRIA2, GRIN1, GRIA1, and HTR1A as key targets mediating the antidepressant activity of L-theanine. These targets are primarily involved in critical pathological processes of depression, including neurotransmitter regulation, glutamatergic synaptic transmission, stress responses, and neurotrophin-related signaling pathways. Molecular docking and molecular dynamics simulations further demonstrated that L-theanine can form stable and energetically favorable complexes with these targets through hydrogen bonding, electrostatic interactions, and hydrophobic interactions, supporting a multi-target synergistic mode of action from both structural and energetic perspectives. Overall, this study provides computational evidence and hypothesis-generating insights into the potential antidepressant mechanisms of L-theanine, which may serve as a reference for future experimental investigations of natural products. However, it should be noted that the present findings are based on computational predictions derived from public databases and simulations, and therefore lack experimental validation. Future research should prioritize validating these predicted mechanisms through cellular assays, animal models, and other experimental approaches to assess the relevance of L-theanine in depression-related interventions and to provide more robust evidence for its therapeutic potential.

## Data Availability

The original contributions presented in this study are included in the article and Supplementary Materials. Further inquiries can be directed to the corresponding authors.

## Supplementary Materials

The following supporting information can be downloaded at: https://www.mdpi.com/article/10.3390/foods15030555/s1, Table S1: Information on 204 Predicted Targets of L-Theanine; Table S2: Depression-Related Targets Identified from Three Databases; Table S3: KEGG Pathway Enrichment Analysis Results; Table S4: Gene Ontology (GO) Enrichment Analysis Results for Biological Process, Cellular Component, and Molecular Function; Table S5: Molecular Docking Results of L-Theanine with Key Targets.

## References

[1] Cosgrove L., Patterson E.H., Bursztajn H.J. (2024). Industry Influence on Mental Health Research: Depression as a Case Example. Front. Med..

[2] Tsoutsi V., Dikeos D., Basta M., Papadakaki M. (2021). Driving Behaviour in Depressed Patients vs Healthy Controls. Eur. J. Public. Health.

[3] Tsoutsi V., Dikeos D., Basta M., Papadakaki M. (2021). Summary of the Clinical Practice Guideline for the Treatment of Depression across Three Age Cohorts. Am. Psychol..

[4] Luxton R., Kyriakopoulos M. (2022). Depression in Children and Young People: Identification and Management NICE Guidelines. Educ. Pract..

[5] Pillinger T., Arumuham A., McCutcheon R.A., D’Ambrosio E., Basdanis G., Branco M., Carr R., Finelli V., Furukawa T.A., Gee S. (2025). The Effects of Antidepressants on Cardiometabolic and Other Physiological Parameters: A Systematic Review and Network Meta-Analysis. Lancet.

[6] Türkmen C., Sacu S., Furukawa Y., de Cates A.N., Schoevers R.A., Kamphuis J., Chevance A., Weisz J.R., Emslie G.J., Strawn J.R. (2025). Side Effect Profile and Comparative Tolerability of Newer Generation Antidepressants in the Acute Treatment of Major Depressive Disorder in Children and Adolescents: Protocol for a Systematic Review and Network Meta-Analysis. BMJ Open.

[7] Chong X., Hou J., He H. (2025). Pharmaceutical Activities of Theanine: A Phytochemical Nutrient. Food Sci. Nutr..

[8] Williams J.L., Everett J.M., D’Cunha N.M., Sergi D., Georgousopoulou E.N., Keegan R.J., McKune A.J., Mellor D.D., Anstice N., Naumovski N. (2019). The Effects of Green Tea Amino Acid L-Theanine Consumption on the Ability to Manage Stress and Anxiety Levels: A Systematic Review. Plant Foods Hum. Nutr..

[9] Moulin M., Crowley D.C., Xiong L., Guthrie N., Lewis E.D. (2024). Safety and Efficacy of AlphaWave^®^ L-Theanine Supplementation for 28 Days in Healthy Adults with Moderate Stress: A Randomized, Double-Blind, Placebo-Controlled Trial. Neurol. Ther..

[10] Moshfeghinia R., Sanaei E., Mostafavi S., Assadian K., Sanaei A., Ayano G. (2024). The Effects of L-Theanine Supplementation on the Outcomes of Patients with Mental Disorders: A Systematic Review. BMC Psychiatry.

[11] Xu W., Song Y., Xiao W., Gong Z. (2023). Regulatory Effects and Mechanisms of L-Theanine on Neurotransmitters via Liver–Brain Axis Under a High Protein Diet. Mol. Neurobiol..

[12] Hidese S., Ota M., Wakabayashi C., Noda T., Ozawa H., Okubo T., Kunugi H. (2016). Effects of Chronic L-Theanine Administration in Patients with Major Depressive Disorder: An Open-Label Study. Acta Neuropsychiatr..

[13] Hopkins A.L. (2008). Network Pharmacology: The next Paradigm in Drug Discovery. Nat. Chem. Biol..

[14] Tuo Y., Lu X., Tao F., Tukhvatshin M., Xiang F., Wang X., Shi Y., Lin J., Hu Y. (2024). The Potential Mechanisms of Catechins in Tea for Anti-Hypertension: An Integration of Network Pharmacology, Molecular Docking, and Molecular Dynamics Simulation. Foods.

[15] Do P.-C., Lee E.H., Le L. (2018). Steered Molecular Dynamics Simulation in Rational Drug Design. J. Chem. Inf. Model..

[16] Hähnke V.D., Kim S., Bolton E.E. (2018). PubChem Chemical Structure Standardization. J. Cheminform..

[17] Jin Z., Zhao H., Luo Y., Li X., Cui J., Yan J., Yang P. (2022). Identification of Core Genes Associated with the Anti-Atherosclerotic Effects of Salvianolic Acid B and Immune Cell Infiltration Characteristics Using Bioinformatics Analysis. BMC Complement. Med. Ther..

[18] Daina A., Michielin O., Zoete V. (2019). SwissTargetPrediction: Updated Data and New Features for Efficient Prediction of Protein Targets of Small Molecules. Nucleic Acids Res..

[19] Tuo Y., Lu X., Song Q., Huang R., Huang J., Sun H., Liao N., Shi Y., Wu L., Lin J. (2025). The Evolution of Gallic Acid in Aged White Tea and Its Potential Anti-Aging Mechanisms: An Integrated Study Combining Network Pharmacology and Computer Simulation. Food Sci. Nutr..

[20] Li J., Zhang H., Xu L., Liu H., Qi C., Wang C., Chen W. (2024). An Integrated Investigation Combining Network Pharmacology and Computer Simulation Dissects the Potential Mechanism of Anti-Obesity of Monascus Pigments (MPs). Food Biosci..

[21] Szklarczyk D., Kirsch R., Koutrouli M., Nastou K., Mehryary F., Hachilif R., Gable A.L., Fang T., Doncheva N.T., Pyysalo S. (2023). The STRING Database in 2023: Protein-Protein Association Networks and Functional Enrichment Analyses for Any Sequenced Genome of Interest. Nucleic Acids Res..

[22] Eberhardt J., Santos-Martins D., Tillack A.F., Forli S. (2021). AutoDock Vina 1.2.0: New Docking Methods, Expanded Force Field, and Python Bindings. J. Chem. Inf. Model..

[23] Lagarias P., Barkan K., Tzortzini E., Stampelou M., Vrontaki E., Ladds G., Kolocouris A. (2020). Correction to Insights to the Binding of a Selective Adenosine A3 Receptor Antagonist Using Molecular Dynamic Simulations, MM-PBSA and MM-GBSA Free Energy Calculations, and Mutagenesis. J. Chem. Inf. Model..

[24] Dash S.G., Kantevari S., Guru S.K., Naik P.K. (2021). Combination of Docetaxel and Newly Synthesized 9-Br-Trimethoxybenzyl-Noscapine Improve Tubulin Binding and Enhances Antitumor Activity in Breast Cancer Cells. Comput. Biol. Med..

[25] Lee J., Hao Nguyen C., Mamitsuka H. (2025). Beyond Rigid Docking: Deep Learning Approaches for Fully Flexible Protein–Ligand Interactions. Brief. Bioinform..

[26] Zhou H., Bie S., Li J., Yuan L., Zhou L. (2022). Comparison on Inhibitory Effect and Mechanism of Inhibitors on SPPO and MPPO Purified from ‘Lijiang Snow’ Peach by Combining Multispectroscopic Analysis, Molecular Docking and Molecular Dynamics Simulation. Food Chem..

[27] Wu W., Zheng Z., Wang Z., Gao C., Liang Y., Zeng W., Sun W. (2024). Profiling of Potential Anti-Diabetic Active Compounds in White Tea: An Integrated Study of Polyphenol-Targeted Metabolomics, Network Pharmacology, and Computer Simulation. Foods.

[28] Chang B., Liu Y., Hu J., Tang Z., Qiu Z., Song Z., Jia A., Zhang Y. (2022). Bupleurum Chinense DC Improves CUMS-Induced Depressive Symptoms in Rats through Upregulation of the CAMP/PKA/CREB Signalling Pathway. J. Ethnopharmacol..

[29] Yüksel C., Öngür D. (2010). Magnetic Resonance Spectroscopy Studies of Glutamate-Related Abnormalities in Mood Disorders. Biol. Psychiatry.

[30] Cai Q., Zhou Z., Luo R., Yu T., Li D., Yang F., Yang Z. (2022). Novel GRIA2 Variant in a Patient with Atypical Autism Spectrum Disorder and Psychiatric Symptoms: A Case Report. BMC Pediatr..

[31] Teyssier J.-R., Ragot S., Chauvet-Gélinier J.-C., Trojak B., Bonin B. (2011). Activation of a ΔFOSB Dependent Gene Expression Pattern in the Dorsolateral Prefrontal Cortex of Patients with Major Depressive Disorder. J. Affect. Disord..

[32] Weder N., Zhang H., Jensen K., Yang B.Z., Simen A., Jackowski A., Lipschitz D., Douglas-Palumberi H., Ge M., Perepletchikova F. (2014). Child Abuse, Depression, and Methylation in Genes Involved with Stress, Neural Plasticity, and Brain Circuitry. J. Am. Acad. Child. Adolesc. Psychiatry.

[33] Verma P., Shakya M. (2021). Machine Learning Model for Predicting Major Depressive Disorder Using RNA-Seq Data: Optimization of Classification Approach. Cogn. Neurodyn..

[34] Xu Z., Chen Z., Shen T., Chen L., Tan T., Gao C., Chen B., Yuan Y., Zhang Z. (2021). The Impact of HTR1A and HTR1B Methylation Combined with Stress/Genotype on Early Antidepressant Efficacy. Psychiatry Clin. Neurosci..

[35] Dai W., Liu J., Qiu Y., Teng Z., Li S., Yuan H., Huang J., Xiang H., Tang H., Wang B. (2022). Gut Microbial Dysbiosis and Cognitive Impairment in Bipolar Disorder: Current Evidence. Front. Pharmacol..

[36] Marques B.L., Lirio P.H.C., Vicente M.A., Unzueta-Larrinaga P., Urigüen L., Campos A.C. (2025). Cannabinoids and Extracellular Vesicles as Potential Biomarkers and Therapeutic Targets in Neuropsychiatric Disorders: A Hypothesis-Driven Review. Pharmaceuticals.

[37] Li R., Huang Z., Luo J., Luo H., Wang W. (2020). Downregulation of the CB1-Mediated Endocannabinoid Signaling Underlies D-Galactose-Induced Memory Impairment. Front. Mol. Neurosci..

[38] Ortiz J.B., Anglin J.M., Daas E.J., Paode P.R., Nishimura K., Conrad C.D. (2018). BDNF and TrkB Mediate the Improvement from Chronic Stress-Induced Spatial Memory Deficits and CA3 Dendritic Retraction. Neuroscience.

[39] Huang Q., Zhang C., Qu S., Dong S., Ma Q., Hao Y., Liu Z., Wang S., Zhao H., Shi Y. (2022). Chinese Herbal Extracts Exert Neuroprotective Effect in Alzheimer’s Disease Mouse Through the Dopaminergic Synapse/Apoptosis Signaling Pathway. Front. Pharmacol..

[40] Vastagh C., Rodolosse A., Solymosi N., Liposits Z. (2016). Altered Expression of Genes Encoding Neurotransmitter Receptors in GnRH Neurons of Proestrous Mice. Front. Cell. Neurosci..

[41] Borruto A.M., Calpe-López C., Spanagel R., Bernardi R.E. (2024). Conditional Deletion of the AMPA-GluA1 and NMDA-GluN1 Receptor Subunit Genes in Midbrain D1 Neurons Does Not Alter Cocaine Reward in Mice. Neuropharmacology.

[42] Tulver K., Bachmann M., Vaht M., Harro J., Bachmann T. (2020). Effects of HTR1A Rs6295 Polymorphism on Emotional Attentional Blink. Acta Neurobiol. Exp..

[43] Li C., Wen R., Liu D., Yan L., Gong Q., Yu H. (2022). Assessment of the Potential of Sarcandra Glabra (Thunb.) Nakai. in Treating Ethanol-Induced Gastric Ulcer in Rats. Based on Metabolomics and Network Analysis. Front. Pharmacol..

[44] Sarker P., Mitro A., Hoque H., Hasan M.N., Nurnabi Azad Jewel G.M. (2023). Identification of Potential Novel Therapeutic Drug Target against Elizabethkingia Anophelis by Integrative Pan and Subtractive Genomic Analysis: An in Silico Approach. Comput. Biol. Med..

[45] Barazorda-Ccahuana H.L., Gómez B., Mas F., Madurga S. (2020). Effect of pH on the Supramolecular Structure of Helicobacter Pylori Urease by Molecular Dynamics Simulations. Polymers.

